# Let's Talk About Mental Health and Mental Disorders in Elite Sports: A Narrative Review of Theoretical Perspectives

**DOI:** 10.3389/fpsyg.2021.700829

**Published:** 2021-06-29

**Authors:** Carolina Lundqvist, Gerhard Andersson

**Affiliations:** ^1^Department of Behavioral Sciences and Learning, Linköping University, Linköping, Sweden; ^2^Department of Biomedical and Clinical Sciences, Linköping University, Linköping, Sweden; ^3^Department of Clinical Neuroscience, Karolinska Institute, Stockholm, Sweden

**Keywords:** elite athlete, sport performance, common mental health disorder, health, well-being

## Abstract

The objective of this article is to discuss: (a) the various theoretical perspectives on mental health and mental health disorders adopted in sport psychology, and (b) how the adoption of these various theoretical perspectives in studies might impact upon the interpretations and conclusions in research about the mental health of participants in elite sports. Well-being as a target construct, holistic models, the single continuum or stage models, and Keyes' dual-continuum model of mental health are described, together with a sports psychiatric view of mental health. The strengths and limitations of various mental health perspectives are discussed. We conclude that mental health is a complex construct and that the sport psychology literature, much like the clinical psychology literature, has struggled to reach a consensus regarding a definition or a feasible approach to investigating mental health. For the researcher, it becomes important to make explicit the underlying theoretical perspective adopted and the operationalization upon which conclusions about elite athletes' mental health are based so that an increased knowledge base with high scientific credibility can be established and consolidated over time.

## Introduction

In recent years, sport psychology researchers have exhibited an almost explosive growth in interest in the investigation of mental health among elite athletes (Kuettel and Larsen, 2020; Poucher et al., 2021). This interest has, at least partly, been stimulated by the mental health movement found in global health-promotion programs calling for greater responsiveness in society overall (e.g., IUHPE, 2018). In a scoping review, Kuettel and Larsen (2020) found that 81% of mental health studies focusing on elite athletes had been published between the years 2013 and 2018. The authors noted that a majority of these studies focused on the assessment of risk factors or various psychological health symptoms possibly related to common mental disorders (Kuettel and Larsen, 2020). Concerns have been expressed in the literature about the high prevalence of mental health issues among elite athletes (e.g., Rice et al., 2016; Reardon et al., 2019), and that stigma in the elite sports culture may decrease help-seeking behaviors and lead sports organizations to depreciate mental health issues as unwelcome “weaknesses” not compatible with high-level sports (e.g., Bauman, 2015; Foskett and Longstaff, 2018; Castaldelli-Maia et al., 2019).

Voices of caution have nevertheless been raised in the sport psychology literature, arguing that the multidimensionality and complexity of separating normal states related to performance issues vs. mental illness or mental disorders may not be being sufficiently considered in today's research (e.g., Uphill et al., 2016; Lebrun and Collins, 2017; Henriksen et al., 2020). For example, increased but transient stress reactions related to challenging sports situations, such as competitions or temporary setbacks, are common and a normal part of the elite sports life (Fletcher and Sarkar, 2012; Turner and Barker, 2013; Martindale et al., 2015). Psychological symptoms associated with the pursuit of the sport may, however, easily be mixed with pathological issues if generic assessments developed for the general population are used on elite athletes without consideration of the psychosocial context in which various symptoms arise (e.g., Lebrun and Collins, 2017; Henriksen et al., 2020; Lundqvist, 2021). Moreover, variations in mood may be linked to the current training load; intense training periods are known to be associated with mood disturbances, but mood usually improves when the training load decrease (Raglin, 2001).

Just as in society at large, mental health literacy and awareness of psychological problems should also be present in elite sports. Deliberate efforts to reduce stigma or to influence decision-makers to take action and increase resources for mental health care among athletes may be justified. These efforts should nevertheless be kept separate from the primary goal of research, which is that the researcher should continuously strive for valid and objective knowledge development to increase the understanding of mental health among elite athletes. Finding a consensus across scholars and disciplines about how to define mental health is difficult and changed societal attitudes toward mental health also change the representation of the mental health construct over time (e.g., Manwell et al., 2015; Bolton and Buhgra, 2021). The lack of consensus has resulted in a wide range of definitions, theories, models, or mental health paradigms being adopted by mental health scholars and practitioners in sport psychology as well as other applied psychological research disciplines (Manwell et al., 2015; Rice et al., 2016; Kuettel and Larsen, 2020). The term “mental health” is sometimes used interchangeably with a desirable mental condition of well-being that may be improved by health factors and proactive efforts (i.e., salutogenesis) and at other times it is used to signal mental ill-being or mental disorders (i.e., pathogenesis; see also Antonowsky, 1979).

The present article aims to discuss various theoretical perspectives of mental health applied in the sport psychology literature, and their impact on evaluations and conclusions about elite athletes' mental health status in research. Both the clinical and sport psychology literature is consulted to provide a historical perspective on the various controversies that still surround these constructs. Theoretical perspectives on positive mental health and approaches targeting non-clinical mental health variations are first presented followed by the psychiatric perspective of mental disorders. Thus, this article especially focuses on exemplifying and discussing (a) the various theoretical perspectives on mental health and mental disorders adopted in clinical psychology and sport psychology, and (b) how the adoption of these various theoretical perspectives in studies might impact upon interpretations and conclusions about the mental health status of participants in elite sports.

## Perspectives on Mental Health in Sport Psychology

The importance of psychological health among elite athletes was most likely first introduced into the sport psychology literature by Morgan (1985), who suggested the Mental Health Model of sports performance, in which psychopathology was examined by means of personality and mood assessments (see also Raglin, 2001). In more recent years, well-being, mental health symptoms, and mental disorders among elite athletes have attracted the interest of researchers (e.g., Lundqvist, 2011; Lundqvist and Raglin, 2015; Gouttebarge et al., 2019; Reardon et al., 2019; Kuettel and Larsen, 2020; Kuettel et al., 2021). Due to the widespread use of the construct of mental health in the sport psychology literature, prominent theoretical models, and orientations will be reviewed below to illuminate the wide scope of meanings that may be attached to this construct.

### Well-Being as a Target Construct of Mental Health

In 1948, the World Health Organization (WHO) brought the first definition of health into force, stating it to be: “complete physical, mental and social well-being and not merely the absence of disease or infirmity” (WHO, 2006, p. 1). In line with the psychological literature in general, the WHO has frequently been referred to over the years when health-related issues are discussed in sport psychology (e.g., Schinke et al., 2017; Kuettel and Larsen, 2020). Although the WHO's first definition should be acknowledged as highlighting health as more than just the absence of physical illness, it has also been criticized over the years; the word “complete” suggests health to be a narrow and almost unattainable state rarely achieved over long periods of life, whereby most people would be classified as unhealthy (Huber et al., 2011). Transferred to an elite sporting context, where daily variations in well-being outcomes are likely to occur as physical and psychological limits are pushed in the striving for athletic development, the adoption of this early definition would be likely to result in conclusions that all elite athletes suffer from ill-being.

A later definition by the WHO (2004) proposed that mental health be defined as “a state of well-being in which the individual realizes his or her own abilities, can cope with the normal stresses of life, can work productively and fruitfully, and is able to make a contribution to his or her community” (p. 12). This definition targets the subjective state of well-being as central to mental health. Assessments of well-being in terms of positive functionality in life (the eudaimonic perspective; Ryff, 2014) and perceived happiness and life satisfaction (the hedonic perspective; Diener, 2009) would accordingly be proper indicators of mental health (cf. Ryan and Deci, 2001; Huta and Ryan, 2010). Models or theoretical approaches commonly adopted as frameworks in well-being studies, for example, the Self-Determination Theory (Ryan and Deci, 2000) and the PERMA model (Seligman, 2011, 2018) closely relate to eudaimonic and hedonic philosophies (Lambert et al., 2015). Application of the well-being view of mental health in research on elite athletes suggests that mental health should be framed as the athlete's psychosocial functionality and ability to nurture individual talents in the lived elite sports environment, subsequently also increasing the probability of the elite athlete regularly experiencing positive affect and life-satisfaction (Lundqvist, 2011, 2021).

Well-being research in sport psychology has been limited by a conceptual lack of clarity in studies that have commonly treated well-being as an undefined “feel-good factor” not related to established theoretical models or frameworks (Lundqvist, 2011; Kuettel and Larsen, 2020). Moreover, the progress of well-being research in sports and other fields of psychology has been limited by a lack of sound measures for assessing well-being (Lundqvist, 2011; Cooke et al., 2016; Linton et al., 2016; Giles et al., 2020). Several sport psychology scholars have nevertheless explicitly studied well-being as a defined target construct of mental health (e.g., Lundqvist, 2011; Lundqvist and Sandin, 2014; Lundqvist and Raglin, 2015; Macdougall et al., 2016, 2019; Kuettel and Larsen, 2020) and well-being is increasingly adopted as an indicator of positive mental health in studies (e.g., Kuettel et al., 2021; McLoughlin et al., 2021). In general, well-being studies have searched for empirical knowledge that can give rise to strategies to maintain, protect, or increase athletes' well-being, both in sports and in life. Thus, the intent in well-being studies on elite athletes has commonly been mental health promotion and a search for protective factors in the sporting context (e.g., Lundqvist and Sandin, 2014; Kuettel and Larsen, 2020; Lundqvist, 2021).

### Holistic Models of Mental Health

In line with the WHO (2004) definition of mental health, holistic perspectives on mental health focus on the whole person and consider the interactional pattern between the person and their environment. This might include a variety of factors; for example, goals, values, functionality/capacity, cultural aspects, and norms in society (e.g., WHO, 2004, 2012; Wittchen et al., 2014; Manwell et al., 2015). Thus, mental health is regarded in these models as a highly complex and multidimensional construct. For example, the biopsychosocial perspective (e.g., Engel, 1980) suggests that biological (e.g., genetic vulnerability, stress reactivity), psychological (e.g., attitudes, cognitions, moods/affects, beliefs, health behaviors, coping skills), and social factors (e.g., family background, social support, environment) interact in intricate patterns to generate various mental health outcomes (Hoffman and Driscoll, 2000). Other holistically oriented approaches found in mental health studies include, for example, ecological models, developmental or life-course models, and quality-of-life perspectives (e.g., Manwell et al., 2015; Reupert, 2017).

The call for a holistic perspective on mental health in elite sports is highly visible in the sport psychology literature; for example, in consensus statements summarizing factors in sports that may relate to elite athletes' mental health, or discussions about the mental health challenges attached to athletes' development, career-transition phases, sports injuries, and dual careers (e.g., Wiese-Bjornstal, 2010; Stambulova et al., 2015, 2020; Wylleman and Rosier, 2016; Schinke et al., 2017; Breslin et al., 2019; Wylleman, 2019; Kuettel and Larsen, 2020; Storm et al., 2021). Purcell et al. (2019) argued for the need to consider the ecological system surrounding the individual athlete when frameworks targeting the promotion of mental health and early intervention or treatment are developed:

In the case of elite athletes, this includes the “microsystem” of coach(es), teammates (where appropriate) and family/loved ones. The wider sporting environment (e.g., the athlete's sport, its rules and governing body) forms the ecosystem, while the role of national and international sporting bodies and the media and broader society form the macrosystem. (p. 3)

Holistic models may indeed provide a comprehensive overview of elite athletes' mental health and the related risk and protective factors in the multifactorial and complex performance-oriented elite sports environment (e.g., Schinke et al., 2017; Henriksen et al., 2020; Stambulova et al., 2020; Storm et al., 2021). From a holistic perspective, variables related to the promotion of mental health as well as the prevention and treatment of mental ill-being could be considered together (Purcell et al., 2019). The complexity and the great number of parameters in need of investigation nevertheless increase the methodological challenges for researchers. For example, various scientific disciplines (e.g., medicine, psychology, sociology) emanating from different research traditions and having different objectives in the study of mental health need to be merged, and the number of plausible mental-health-related variables to consider may be almost innumerable (e.g., Thiel et al., 2015). The search for a holistic understanding of mental health may therefore also pose a risk of reduced precision and internal validity in studies that provide complex or overarching models or frameworks that might be difficult to evaluate empirically.

### Single Continuum and Stage Models of Mental Health

Continuum models of mental health have become increasingly promoted in the sport psychology literature among researchers studying elite athletes' mental health (e.g., Schinke et al., 2017; Moesch et al., 2018; Purcell et al., 2019). These continuum models rest on the idea that individuals can move bidirectionally along a single mental health–mental disorder continuum (e.g., Haggerty and Mrazek, 1994; Chen et al., 2020). Mental health indicators usually range from (a) normal variations in mood and psychological and social activity, through (b) normal emotional or behavioral reactions to life-situations (e.g., being nervous, sad, decreased social activity), to (c) increased levels of psychological harm or injury (e.g., anxiety, reduced performance, pervasive sadness, difficulties concentrating, social withdrawal), and finally (d) mental illness in terms of diagnosable psychiatric conditions (e.g., Chen et al., 2020). Some models also suggest interventions aimed at risk-reduction for mental illness symptoms (i.e., prevention) at one extreme of the continuum and treatment and relapse prevention conducted in clinical care at the opposite end (Haggerty and Mrazek, 1994; Purcell et al., 2019). Closely related to single-continuum models are stage models, in which the heterogeneity of mental health or disorders are displayed stepwise; for example, from wellness to distress and thereafter to clinical disorders of various severities (Patel, 2017). Overall, these models target various symptoms and generally promote the use of self-care and health-related actions (e.g., sleep, nutrition, recovery, seeking low-intensity support from family and friends) when early and mild symptoms appear, and appropriate psychological interventions or psychiatric care in cases where symptoms are severe and in need of clinical treatment (Patel, 2017; Chen et al., 2020).

Single-continuum models as part of frameworks for mental health among elite athletes have been argued as useful in elite sports to account for the great variation in expressions of psychological health and levels of functioning among athletes beyond categorical perspectives (e.g., Schinke et al., 2017; Purcell et al., 2019). While continuum models might be a viable approach to improving our understanding of mental health among elite athletes, their adoption in studies on elite athletes also pose challenges related to the interpretation of symptoms. This is because emotion-related symptoms (e.g., distress, anxiety, stress), which occur temporarily as natural and appropriate consequences of elite athletes' sporting pursuits, may easily be misinterpreted as failing mental health in cross-sectional studies (e.g., Henriksen et al., 2020; Lundqvist, 2021). Thus, these assessments should be complemented with additional information obtained, for example, by the use of clinical interviews. Moreover, the learning experiences that athletes gain from exposure to moderately challenging life and sporting situations are often linked with momentary adverse emotional responses. For some athletes, such experiences might act to enable long-term psychological resources and increase levels of resilience, helping them to bounce back and stay mentally healthy in future situations (Seery et al., 2010; Collins and MacNamara, 2012; Sarkar et al., 2015; Henriksen et al., 2020). Thus, in the absence of health-related information other than self-reports of emotion-related symptoms, continuum models do not provide any guidance as to whether reported symptoms should be regarded as natural reactions to sports or early signs of mental health concerns or disorders. These models, therefore, leave interpretations highly open to the researchers' framing of symptoms. Based on the nature of elite sports and the basic idea underlying single-continuum models, it is to be expected that, if assessed over time, many elite athletes would move back and forth along the mental health continuum without necessarily being at risk of developing clinically relevant concerns or mental disorders in need of treatment.

### Keyes' Dual Continuum-Model to Explain Mental Health

The human emotional repertoire is known to include both positive (e.g., well-being) and negative (e.g., sadness, unhappiness, anxiety) emotional states, and a fully lived life will also be very likely to include occasional periods when functionality and productively are temporarily lacking (Hagen, 2011; Galderisi et al., 2015). Keyes (2002, 2003) noted that a one-dimensional reliance on the assessment of either well-being or ill-being to indicate mental health may be inadequate for a comprehensive understanding of complete mental health, and therefore introduced a two-dimensional approach called the dual-continuum model. The dual-continuum model suggests that mental health consists of a mental illness continuum and a mental health continuum, where symptoms on each dimension may range independently from high to low. The two dimensions are viewed as related but essentially distinct. According to this perspective, assessments of the symptoms of mental health (i.e., emotional, psychological, and social well-being) and symptoms of mental illness should be performed simultaneously to obtain a comprehensive understanding of the individual's complete mental health status. Crossing the dimensions results in three plausible states of mental health: languishing, moderate mental health, and flourishing (Keyes, 2002, 2003, 2005). Complete mental health would be indicated by low levels or the complete absence of mental illness symptoms and high levels of mental health symptoms. Mental illness would be indicated by the opposite pattern, with low levels or a complete absence of well-being symptoms combined with high levels of ill-being symptoms (Keyes, 2003).

Based on the complex nature of elite sports, where a range of health-related symptoms may be reported, some scholars (e.g., Uphill et al., 2016) have argued in favor of theoretically adopting the dual-continuum model to improve our understanding of elite athletes' mental health. Nevertheless, the use of Keyes' (2002) model imposes increased challenges when interpreting athletes' mental health status. For example, Van Slingerland et al. (2018) found some student-athletes to maintain moderate to high levels of mental health functioning and flourishing, while at the same time also reporting being diagnosed with a mental disorder. Similar complexities in interpretations of mental health and mental ill-being continuums were reported by Durand-Bush et al. (2015), who found that athletes reported low mental health functioning and moderate levels of stress but also moderate levels of self-regulation capacity and moderately high psychological well-being, suggesting a positive adjustment. Adopting the dual-continuum model as a theoretical framework, Kuettel et al. (2021) performed latent profile analysis to identify mental health profiles based on athletes, anxiety, depression, and well-being scores. In a sample of 612 Danish elite athletes, the majority (64.2%) of the athletes were profiled as flourishing, approximately a third (29.3%) as having a moderate mental health profile, and a minority of the sample (6.5%) as languishing. Moreover, athletes classified into the various mental health profiles differed significantly in self-reported scores of protective factors (social support, sleep, perception of sport environment) and risk-factors (perceived stressors, workload; Kuettel et al., 2021). Thus, by adopting the dual-continuum model, “the absence of mental health does not imply the presence of mental illness, and the presence of mental illness, does not imply the absence of mental health” (Uphill et al., 2016, p. 3). Multiple assessments targeting both positive mental health and mental ill-being may provide extended information to the interpretation of elite athletes' complete mental health.

## Mental Disorders

During the past few years, mental health studies among elite athletes have increasingly assumed a biomedical model based on medicine and psychiatry (e.g., Markser, 2011; Bär and Markser, 2013; Ströhle, 2019). The psychiatric view is also visible in the International Olympic Committee's (IOC) approach toward protecting mental health in elite athletes (Reardon et al., 2019; Gouttebarge et al., 2021). Sport psychiatry adopts psychopharmaceutical and psychotherapeutic approaches to treatments when mental disorders are diagnosed among athletes. At an overarching level, the procedure of diagnosing mental disorders acts to separate pathological conditions from what could be regarded as non-clinical mental health variations among populations (Amoretti and Lalumera, 2019). The sports psychiatric perspective adopts the today's dominant diagnostic tools, which are the Diagnostic and Statistical Manual of Mental Disorders-5 (DSM-5; American Psychiatric Association, 2013) and the International Statistical Classification of Diseases and Related Health Problems-11 (ICD-11; cf. Pocai, 2019).

### General Controversies Surrounding Mental Disorders

Although seldom explicitly discussed or problematized to any great extent in the sport psychology literature, the definition of a mental disorder and its classification is still a matter of debate and controversy (e.g., Bell, 1994; Andersson and Ghaderi, 2006; Bolton, 2013; Frances, 2013; Poucher et al., 2021). The deductive, top-down approach of classifying behavioral problems into diagnostic categories has been criticized by clinical psychology scholars adopting alternative approaches, for example functional analysis used in cognitive-behavioral therapy, which to a greater extent target the idiographic and contextual perspective of the person (Andersson and Ghaderi, 2006). The definition of mental disorders has also varied over time, with changed inclusion/exclusion criteria or classification systems due to scientific progress, but also due to the dominant political and social interests/needs of different periods (Kinderman et al., 2017; Amoretti and Lalumera, 2019). Changed boundary criteria for mental disorder diagnoses across editions of the DSM have triggered debates in the literature (Bolton, 2013; Frances, 2013; Sweet and Decoteau, 2018). For example, the bereavement criterion in DSM-IV prevented people in grief from being diagnosed with depression, but this criterion was excluded from the fifth edition of the DSM manual, a choice that has been criticized for increasing the over-diagnosing and pathologization of normal life reactions (e.g., Frances, 2013; Wakefield, 2013, 2016; Paris, 2015).

What is considered to be the value of diagnoses of mental disorders for a clinician or sports psychologist might also be different from the perspective of a researcher, and researchers might vary depending on their philosophical orientation and the objectives of their research (Andersson and Ghaderi, 2006; Heckers, 2015). A diagnosis of a mental disorder may be explained as: (a) something real, biological, and independent of human beliefs, perceptions, or activities (realism; Kendler, 2016), (b) a concept that changes based on the present societal norms and ideas of what is regarded as normal and deviant, which may subsequently loop back to how people identify themselves through these concepts and the language used to describe themselves (e.g., Hacking, 1998, 2004; Brinkmann, 2005; Kendler, 2016; constructivism), and (c) a tool that helps the clinician to organize complex clinical observations, to ease communication between caregivers and to guide treatment as well as make predictions about progress and outcomes (pragmatism; Kendell and Jablensky, 2003; Kendler, 2016). Thus, the definitions of mental disorders and various diagnoses are far from being a unified set of concepts.

### Mental Disorders and Sport Psychiatry

In the elite sport literature, a rapidly growing number of studies have chosen to adopt a sport psychiatry language to describe mental health. Thus, they have sought to investigate the prevalence among elite athletes of self-reported symptoms related to mental health disorders. Gouttebarge et al. (2019), for example, included 37 studies in their systematic review and meta-analysis, and reported the prevalence of current and former elite athletes' self-reported symptoms of alcohol misuse, distress, sleep disturbances, and anxiety/depression. Rice et al. (2019) identified a total of 61 studies eligible for a systematic review of elite athletes' anxiety symptoms and disorders. In general, studies have suggested that the prevalence levels of symptoms related to common mental health disorders are comparable with levels found in non-athlete populations (Gorczynski et al., 2017; Rice et al., 2019). Great variations in reported prevalence levels can nevertheless be found in research conducted to date, with a wide range of screening tools being used to estimate various mental health symptoms and disorders among elite athletes (e.g., Gouttebarge et al., 2019; Reardon et al., 2019; Rice et al., 2019). Golding et al. (2020), for example, found variations from 7 to 34% across studies investigating prevalence levels of depression among high-level elite athletes. These variations were at least partly explained by the studies' adoption of diverse depression assessments in the absence of clinical interviews to confirm a diagnosis of depression (Golding et al., 2020).

The definitions and assessments adopted by researchers are known to impact upon reported prevalence in populations (e.g., Migliavaca et al., 2020). For example, Schinnar et al. (1990) compared 17 definitions of mental disorders and found that estimates of the prevalence of severe mental illness ranged from 4 to 88%, contingent upon which definition was adopted in studies. Thus, the operationalization of mental health symptoms and disorders in studies, and how broad or narrow are the criteria used to determine whether symptoms constitute a health concern or not, will impact upon the prevalence levels reported in various athlete populations. Thus, the scientific choices when investigating symptoms and diagnoses of mental disorders among elite athletes provide room for variation and the scientific goals of researchers, the philosophical perspective on mental disorders adopted, and the choice of assessment should be visible and explained in all studies.

## General Discussion

Like other subdisciplines of psychology, the sport psychology literature has struggled to find consensus on a definition or a feasible approach to investigate mental health constructs among elite athletes. Various theoretical perspectives adopted by mental health researchers may also impose strengths and limitations in results obtained in studies. What is considered to be mental health or a lack of mental health will vary in studies depending on the definition, theoretical perspective, and assessment chosen by researchers.

### The First Phase of Research on Mental Health Concerns in Elite Sports

Considering the increasing number of articles published in recent years, the view that elite athletes will be immune to mental health concerns seems to have been, until recently, a prevailing view among many researchers, practitioners, and sports organizations (Bär and Markser, 2013; Bauman, 2015). The current evidence base on elite athletes' mental health is dominated by the clinical and sports psychiatric perspective and to an increasing extent also a holistic or continuum perspectives (Schinke et al., 2017; Gouttebarge et al., 2019; Kuettel and Larsen, 2020; Stambulova et al., 2020; Rice et al., 2021). Several sport psychology organizations have responded to the mental health movement by producing consensus or position statements discussing mental health symptoms and disorders in the context of elite sports (Schinke et al., 2017; Moesch et al., 2018; Van Slingerland et al., 2018; Gorczynski et al., 2019; Reardon et al., 2019). Thus, great efforts have been made on research aiming to raise awareness that mental health concerns can also be present among elite athletes, enabling the question to be put on the sport political agenda. This first phase of research should be acknowledged for uncovering the reality that elite athletes are not superheroes but human beings, and therefore are not immune to mental health concerns. However, a shotgun approach has seemingly been adopted to collect data by the application of a wide range of general and clinically oriented screening instruments among widely distributed samples of elite athletes. From a scientific standpoint, and in terms of continued knowledge development, conclusions about elite athletes' mental health should not be running ahead of valid empirical data on the subject. Definitions of mental health, research orientations, and methodological quality should therefore be considered very carefully when evaluating and interpreting the results on elite athletes' mental health drawn from research conducted during this initial phase.

### Toward a Second Phase of Mental Health Research in Elite Sports

A stress–mental health vulnerability hypothesis has been assumed in the sport psychology literature, implying that elite athletes who are exposed to a great number of distinct and elite sport-related stressors are more vulnerable to mental health concerns than non-athletes not exposed to these stressors (e.g., Arnold and Fletcher, 2012; Gorczynski et al., 2017). A direct relationship between exposure to elite sports stressors and mental health concerns is, however, challenged in available research, which indicates comparable levels of symptoms reported among elite athletes and non-athlete populations (e.g., Gulliver et al., 2015; Rice et al., 2016; Gorczynski et al., 2017; Gouttebarge et al., 2019). Research conducted in the general clinical psychological literature confirms that the relationship between stress and mental health disorders (e.g., depression) is highly complex, with several plausible biopsychosocial factors mediating the relationships (Hammen, 2015). In addition, psychological sciences emphasize the interplay between various biological, psychological, and socio-environmental variables, and their interplay, to a greater extent than the biomedical view of mental health. Thus, a dimensional rather than a categorical or diagnostic view may be preferable when searching for an understanding of what could be considered normal or abnormal behaviors (Wittchen et al., 2014; Rice et al., 2021).

Scholars have cautioned that the categorization and labeling of mental health into psychiatric diagnoses may pose a risk of an exaggerated individualization and pathologization of problems that may actually be related to social and structural elements in the person's life (Kvist Lindholm and Wickström, 2020). Thus, moving toward the second phase of research on mental health among elite athletes, mediating biopsychosocial factors (e.g., learning history through life, genetic vulnerability, sleep, nutrition, mental health literacy, coping repertoire, resilience, social support, environmental, and cultural factors within and outside elite sports) that might explain with greater precision the various relationships between stress exposure and mental health outcomes in the elite sports context need further investigation by empirical studies. A greater use of qualitative methods in mental health research would also enable a deepened understanding of the lived experiences of elite athletes together with a broader insight into various protective and risk-factors for mental health that elite athletes may perceive during their careers. Several theoretical perspectives on mental health (e.g., holistic perspectives, single or dual-continuum models) previously exemplified in this article focus to a greater extent on health promotion (salutogenesis) than illness and treatment (pathogenesis). Some empirical studies have also found elite athletes to report a better quality of life than normal populations both during and after their elite careers (Filbay et al., 2017, 2019; Bullock et al., 2020). Plausible explanations for these findings may be that the learning experiences and skills acquired through participation in elite sports build psychological resources (e.g., resilience, awareness of the body's capabilities, self-management skills) that act as protective factors in the long term (e.g., Bullock et al., 2020). The use of alternative theoretical approaches than the biomedical model may therefore provide alternative understandings of mental health expressions or outcomes and provide insights into useful support strategies for elite athletes (cf. Lundqvist, 2021). Contemporary scholars within this research field are increasingly also promoting the development of extended psychosocial support systems around athletes, focusing on a sustainable overall situation to help athletes maintain their mental health and to develop over time, including during challenging career phases (Stambulova et al., 2015, 2020; Wylleman and Rosier, 2016; Breslin et al., 2019; Purcell et al., 2019; Lundqvist, 2020, 2021; Storm et al., 2021). Arguably, from both a mental health and performance perspective, there are benefits in proactively supporting high-level athletes to develop the psychosocial resources to remain functional with experienced quality of life both during and after their elite sporting careers. A majority of research has so far been conducted in Western countries, with Europe and Oceania prominent among these, and protective factors are still less studied (Kuettel and Larsen, 2020). Elite-sports systems and cultures may differ greatly across countries. Future researchers on this topic are therefore encouraged to expand the scope of nationalities and continents studied to increase the knowledge of cross-cultural differences and similarities in mental health expressions, needs, and the development of support systems around the world.

### Summarizing Conclusions

Mental health is a highly complex construct and, based on the history from other research areas, it seems unlikely that consensus will be reached on a uniform definition to be used in elite sporting contexts. To establish an increased knowledge base with high scientific credibility over time, it thus becomes important to make explicit the underlying theoretical perspective adopted and the operationalization on which conclusions about elite athletes' mental health are based. Various theoretical perspectives and methodological approaches to mental health inflict strengths and limitations but also bring different views on mental health that may act to increase a more holistic understanding of how mental health among elite athletes can be promoted in their lived context.

## Author Contributions

CL initiated and wrote the manuscript. GA critically reviewed and revised the manuscript for intellectual content before submission. The authors discussed and agreed upon the main messages during the preparation of the paper. All authors contributed to the article and approved the submitted version.

## Conflict of Interest

The authors declare that the research was conducted in the absence of any commercial or financial relationships that could be construed as a potential conflict of interest.

## References

[B1] American Psychiatric Association (2013). Diagnostic and Statistical Manual of Mental Disorders, 5th Edn. 10.1176/appi.books.9780890425596

[B2] Amoretti C. M. Lalumera E. (2019). A potential tension in DSM-5: the general definition of mental disorder versus some specific diagnostic criteria. J. Med. Philos. 44, 85–108. 10.1093/jmp/jhy001 29850842

[B3] Andersson G. Ghaderi A. (2006). Overview and analysis of the behaviourist criticism of the Diagnostic and Statistical Manual of Mental Disorders (DSM). J. Clin. Psychol. 10, 67–77. 10.1080/13284200600690461

[B4] Antonowsky A. (1979). Stress, Health, and Coping. San Francisco, CA: Jossey-Bass Inc.

[B5] Arnold R. Fletcher D. (2012). A research synthesis and taxonomic classification of the organizational stressors encountered by sport performers. J. Sport. Exerc. Psychol. 34, 397–429. 10.1123/jsep.34.3.397 22691400

[B6] Bär K. J. Markser V. Z. (2013). Sport specificity of mental disorders: the issue of sport psychiatry. Eur. Arch. Psychiatry Clin. Neurosci. 263, 205–210. 10.1007/s00406-013-0458-4 24091603

[B7] Bauman J. (2015). The stigma of mental health in athletes: are mental toughness and mental health seen as contradictory in elite sport? Br. J. Sports Med. 50, 135–136. 10.1136/bjsports-2015-095570 26626270

[B8] Bell C. (1994). Philosophical perspectives on psychiatric diagnostic classification. J. Am. Med. Assoc. 272, 1794–1796. 10.1001/jama.1994.03520220090039

[B9] Bolton D. (2013). Overdiagnosis problems in the DSM-IV and the new DSM-5: can they be resolved by the distress-impairment criterion. Can. J. Psychiatry 58, 612–617. 10.1177/070674371305801106 24246431

[B10] Bolton D. Buhgra D. (2021). Changes in society and young people's mental health. Int. Rev. Psychiatry 33, 154–161. 10.1080/09540261.2020.1753968 32347134

[B11] Breslin G. Smith A. Donohue B. Donnelly P. Shannon S. Jane Haughey T.. (2019). International consensus statement on the psychosocial and policy-related approaches to mental health awareness programmes in sport. BMJ Open Sport Exerc. Med. 5:e000585. 10.1136/bmjsem-2019-000585 31673406 PMC6797268

[B12] Brinkmann S. (2005). Human kinds and looping effects in psychology: foucauldian and hermeneutic perspectives. Theory Psychol. 15, 769–791. 10.1177/0959354305059332

[B13] Bullock G. S. Collins G. S. Peirce N. Arden N. K. Filbay S. R. (2020). Health-related quality of life and flourishing in current and former recreational and elite cricketers. Health Qual. Life Outcomes 18:41. 10.1186/s12955-020-01301-7 32093738 PMC7038545

[B14] Castaldelli-Maia J. M. Gallinaro J. G. D. M. E. Falcão R. S. Gouttebarge V. Hitchcock M. E. Hinline B.. (2019). Mental health symptoms and disorders in elite athletes: a systematic review on cultural influencers and barriers to athletes seeking treatment. Br. J. Sports Med. 53, 707–721. 10.1136/bjsports-2019-100710 31092400

[B15] Chen S. P. Chang W. P. Stuart H. (2020). Self-reflection and screening mental health on Canadian campuses: validation of the mental health continuum model. BMC Psychol. 8:76. 10.1186/s40359-020-00446-w 32727614 PMC7391623

[B16] Collins D. MacNamara A. (2012). The rocky road to the top: why talent needs trauma. Sports Med. 42, 907–914. 10.1007/BF03262302 23013519

[B17] Cooke P. J. Melchert T. P. Connor K. (2016). Measuring well-being: a review of instruments. Counsel. Psychol. 44, 730–757. 10.1177/0011000016633507

[B18] Diener E. (2009). “Subjective well-being”, in Social Indicators of Research Series. The Science of Well-Being, Diener E.ed. (New York, NY: Springer Science-Business Media B.V.), 11–58.

[B19] Durand-Bush N. McNeil K. Harding M. Dobransky J. (2015). Investigating stress, psychological well-being, mental health functioning, and self-regulation capacity among university undergraduate students: is this population optimally functioning? Can. J. Counsell. Psychother. 49, 253–274. Available online at: https://cjc-rcc.ucalgary.ca/article/view/61066

[B20] Engel G. L. (1980). The clinical application of the biopsychosocial model. Am. J. Psychiatry 137, 535–544. 10.1176/ajp.137.5.535 7369396

[B21] Filbay S. R. Bishop F. Peirce N. Jones M. E. Arden N. K. (2017). Common attributes in retired professional cricketers that may enhance or hinder quality of life after retirement: a qualitative study. BMJ Open 7:e016541. 10.1136/bmjopen-2017-016541 28751489 PMC5642649

[B22] Filbay S. R. Pandya T. Thomas B. McKay C. Adams J. Arden N. (2019). Quality of life and life satisfaction in former athletes: a systematic review and meta-analysis. Sports Med. 49, 1723–1738. 10.1007/s40279-019-01163-0 31429036 PMC6789047

[B23] Fletcher D. Sarkar M. (2012). A grounded theory of psychological resilience in Olympic champions. Psychol. Sport. Exerc. 13, 669–678. 10.1016/j.psychsport.2012.04.007

[B24] Foskett R. L. Longstaff F. (2018). The mental health of elite athletes in the United Kingdom. J. Sci. Med. Sport. 21, 765–770. 10.1016/j.jsams.2017.11.016 29289498

[B25] Frances A. (2013). Saving Normal. An Insider's Revoult Against Out-Control Psychiatric Diagnosis, DSM-5, Big Pharma, and the Medicalization of Ordinar. New York, NY: William Morrow Company.

[B26] Galderisi S. Heinz A. Katrup M. Beezhold J. Sartorius N. (2015). Toward a new definition of mental health. World Psychiatry 14, 231–233. 10.1002/wps.20231 26043341 PMC4471980

[B27] Giles S. Fletcher D. Arnold R. Ashfield A. Harrison J. (2020). Measuring well-being in sport performers: where are we now and how do we progress? Sports Med. 50, 1255–1270. 10.1007/s40279-020-01274-z 32103451 PMC7305091

[B28] Golding L. Gillingham R. G. Perera N. K. P. (2020). The prevalence of depressive symptoms in high-performance athletes: a systematic review. Phys. Sportsmed. 48, 247–258. 10.1080/00913847.2020.1713708 31964205

[B29] Gorczynski P. F. Coyle M. Gibson K. (2017). Depressive symptoms in high-performance athletes and non-athletes: a comparative meta-analysis. Br. J. Sports Med. 51, 1348–1354. 10.1136/bjsports-2016-096455 28254747

[B30] Gorczynski P. F. Gibson K. Thelwell R. Papathomas A. Harwood C. Kinnafick F. (2019). The BASES expert statement on mental health literacy in elite sport. Sport Exerc. Sci. 59, 6–7. Available online at: https://www.bases.org.uk/imgs/7879_bas_expert_statement__pages_735.pdf

[B31] Gouttebarge V. Bindra A. Blauwet C. Campriani N. Currie A. Engebretsen L.. (2021). International Olympic Committee (IOC) Sport Mental Health Assessment Tool 1 (SMHAT-1) and Sport Mental Health Recognition Tool 1 (SMHRT-1): towards better support of athletes' mental health. Br. J. Sports Med. 55, 30–37. 10.1136/bjsports-2020-102411 32948518 PMC7788187

[B32] Gouttebarge V. Castaldelli-Maia J. M. Gorczynski P. Hainline B. Hitchcock M. E. Kerkhoffs G. M.. (2019). Occurrence of mental health symptoms and disorders in current and former elite athletes: a systematic review and meta-analysis. Br. J. Sports Med. 53, 700–706. 10.1136/bjsports-2019-100671 31097451 PMC6579497

[B33] Gulliver A. Griffiths K. M. Mackinnon A. Batterham P. J. Stanimirovic R. (2015). The mental health of Australian elite athletes. J. Sci. Med. Sport. 18, 255–261. 10.1016/j.jsams.2014.04.006 24882147

[B34] Hacking I. (1998). Mad Travelers: Reflections on the Reality of Transient Mental Disease. Charlottesville, VA: University Press of Virginia.

[B35] Hacking I. (2004). Between Michel Foucault and Erving Goffman: between discourse in the abstract and faceto-face interaction. Econ. Soc. 333, 277–302. 10.1080/0308514042000225671

[B36] Hagen E. H. (2011). Evolutionary theories of depression: a critical review. Can. J. Psychiatry 56, 716–726. 10.1177/070674371105601203 22152640

[B37] Haggerty R. J. Mrazek P. J. (1994). Reducing Risks for Mental Disorders: Frontiers for Preventive Intervention Research. Washington, DC: National Academies Press. 25144015

[B38] Hammen C. L. (2015). Stress and depression: old questions, new approaches. Curr. Opin. Psychol. 4, 80–85. 10.1016/j.copsyc.2014.12.024

[B39] Heckers S. (2015). The value of psychiatric diagnoses. JAMA Psychiatry 72, 1165–1166. 10.1001/jamapsychiatry.2015.2250 26560736

[B40] Henriksen K. Schinke R. Moesch K. McCann S. Parham W. D. Larsen C. W.. (2020). Consensus statement on improving the mental health of high performance athletes. Int. J. Sport. Exerc. Psychol. 18, 553–560, 10.1080/1612197X.2019.1570473

[B41] Hoffman M. A. Driscoll J. M. (2000). “Health promotion and disease prevention: a concentric biopsychosocial model of health status,” in I Handbook of Counseling Psychology, eds. S. D. Brown and R. W. Lent (Hoboken, NJ: John Wiley and Sons Inc), 532–567.

[B42] Huber M. Knottnerus A. J. Green L. van der Horst H. Jadad A. R. Kromhout D.. (2011). How should we define health? BMJ 343:d4163. 10.1136/bmj.d4163 21791490

[B43] Huta V. Ryan R. M. (2010). Pursuing pleasure or virtue: the differential and overlapping well-being benefits of hedonic and eudaimonic motives. J. Happiness Stud. 11, 735–762. 10.1007/s10902-009-171-4

[B44] IUHPE (2018). Position Statement on health literacy: a practical vision for a health literate world. Glob. Health Promot. 25, 79–88. 10.1177/1757975918814421

[B45] Kendell R. Jablensky A. (2003). Distinguishing between the validity and utility of psychiatric diagnoses. Am. J. Psychiatry 160, 4–12. 10.1176/appi.ajp.160.1.4 12505793

[B46] Kendler K. S. (2016). The nature of psychiatric disorders. World Psychiatry 15, 5–12. 10.1002/wps.20292 26833596 PMC4780286

[B47] Keyes C. L. M. (2002). The mental health continuum: from languishing to flourishing in life. J. Health Soc. Behav. 43, 207–222. 10.2307/3090197 12096700

[B48] Keyes C. L. M. (2003). “Complete mental health: an agenda for the 21st century” in Flourishing: Positive Psychology and the Life Well-Lived, eds C. L. M. Keyes and J. Haidt (Washington, DC: American Psychological Association), 293–312.

[B49] Keyes C. L. M. (2005). Mental illness and/or mental health? Investigating axioms of the complete state model of health. J. Consult. Clin. Psychol. 73, 539–548. 10.1037/0022-006X.73.3.539 15982151

[B50] Kinderman P. Allsopp K. Cooke A. (2017). Responses to the publication of the American Psychiatric Association's DSM-5. J. Hum. Psychol. 57, 625–649. 10.1177/0022167817698262

[B51] Kuettel A. Larsen C. H. (2020). Risk and protective factors for mental health in elite athletes: a scoping review. J. Sport Exerc. Psychol. 13, 231–265. 10.1080/1750984X.2019.1689574

[B52] Kuettel A. Pedersen A. K. Larsen C. H. (2021). To flourish or languish, that is the question: exploring the mental health profiles of Danish elite athletes. Psychol. Sport Exerc. 52:101837. 10.10.1016/j.psychsport.2020.101837

[B53] Kvist Lindholm S. Wickström A. (2020). “Looping effects” related to young people's mental health: how young people transform the meaning of psychiatric concepts. Glob. Stud. Child. 10, 26–38. 10.1177/2043610619890058

[B54] Lambert L. Passmore H. A. Holder M. D. (2015). Foundational frameworks of positive psychology: mapping well-being orientations. Can. Psychol. 56, 311–321. 10.1037/cap0000033

[B55] Lebrun F. Collins D. (2017). Is elite sport (really) bad for you? Can we answer the question? Front. Psychol. 8:324. 10.3389/fpsyg.2017.00324 28316585 PMC5334321

[B56] Linton M. J. Dieppe P. Medina-Lara A. (2016). Review of 99 self-report measures for assessing well-being in adults: exploring dimensions of well-being and developments over time. BMJ Open 6:e010641. 10.1136/bmjopen-2015-010641 27388349 PMC4947747

[B57] Lundqvist C. (2011). Well-being in competitive sports – the feel-good factor? A review of conceptual considerations in well-being research. Int. J. Sport Exerc. Psychol. 4, 109–128. 10.1080/1750984X.2011.584067

[B58] Lundqvist C. (2020). Ending an elite sports career: case report of behavioral activation as an evidence-based intervention with a former Olympic athlete developing depression. Sport Psychol. 34, 329–336. 10.1123/tsp.2019-0152

[B59] Lundqvist C. (2021). “Well-being and quality of life”, in *Stress, Well-Being and Performance in Sport*, eds D. Fletcher and R. Arnold (London: Routledge), 131–147.

[B60] Lundqvist C. Raglin J. (2015). The relationship of basic need satisfaction, motivational climate and personality to well-being and stress patterns among elite athletes: an explorative study. Motiv. Emot. 39, 237–246. 10.1007/s11031-014-9444-z

[B61] Lundqvist C. Sandin F. (2014). Well-being in elite sport: dimensions of hedonic and eudaimonic well-being among elite orienteers. Sport Psychol. 28, 245–254. 10.1123/tsp.2013-0024

[B62] Macdougall H. O'Halloran P. Sherry E. Shields N. (2019). A pilot randomised controlled trial to enhance well-being and performance of athletes in para sports. Eur. J. Adapt. Phys. Act. 12, 1–19. 10.5507/euj.2019.006

[B63] Macdougall H. O'Holloran P. Sherry E. Shields N. (2016). Needs and strengths of Australian Para athletes: identifying the subjective, psychological, social, and physical health and well-being. Sport Psychol. 30, 1–12. 10.1123/tsp.2015-0006

[B64] Manwell L. A. Barbic S. P. Roberts K. Durisko Z. Cheolsoon L. Ware E.. (2015). What is mental health? Evidence towards a new definition from a mixed methods multidisciplinary international survey. BMJ Open 5:e007079. 10.1136/bmjopen-2014- 007079 26038353 PMC4458606

[B65] Markser V. Z. (2011). Sport psychiatry and psychotherapy. Mental strains and disorders in professional sports. Challenge and answer to societal changes. Eur. Arch. Psychiatry Clin. Neurosci. 261, 182–185. 10.1007/s00406-011-0239-x 21901268

[B66] Martindale A. Collins D. Richard H. (2015). Is elite sport (still) good for you? A response to the reply. Sport Exerc. Psychol. Rev. 11, 87–91. Available online at: https://www.research.ed.ac.uk/en/publications/its-good-to-talk-is-elite-sport-still-good-for-you-a-response-to-

[B67] McLoughlin E. Fletcher D. Slavich G. M. Arnold R. Moore L. J. (2021). Cumulative lifetime stress exposure, depression, anxiety, and well-being in elite athletes: a mixed-method study. Psychol. Sport Exerc. 52:101823. 10.1016/j.psychsport.2020.101823 33281503 PMC7710337

[B68] Migliavaca C. B. Stein C. Colpani V. Munn Z. Falavigna M. (2020). Quality assessment of prevalence studies: a systematic review. J. Clin. Epidemiol. 127, 59–68. 10.1016/j.jclinepi.2020.06.039 32679313

[B69] Moesch K. Kenttä G. Kleinert J. Quignon-Fleur C. Cecil S. Bertollo M. (2018). FEPSAC position statement: mental health disorders in elite athletes and models of service provision. Psychol. Sport Exerc. 38, 61–71. 10.1016/j.psychsport.2018.05.013

[B70] Morgan W. P. (1985). “Selected psychological factors limiting performance: a mental health model,” in Limits of Human Performance, eds D. H. Clarke and H. M. Eckert (Champaign, IL: Human Kinetics), 70–80.

[B71] Paris J. (2015). The Intelligent Clinician's Guide to DSM-5®, 2nd Edn. New York, NY: Oxford University Press.

[B72] Patel V. (2017). Talking sensibly about depression. PLoS Med. 14:e1002257. 10.1371/journal.pmed.1002257 28376089 PMC5380305

[B73] Pocai B. (2019). The ICD-11 has been adopted by the World Health Assembly. World Psychiatry 18, 371–372. 10.1002/wps.20689 31496092 PMC6732675

[B74] Poucher Z. A. Tamminen K. A. Kerr G. Karney J. (2021). A commentary on mental health research in elite sport. J. Appl. Sport Psychol. 33, 60–82. 10.1080/10413200.2019.1668496

[B75] Purcell R. Gwyther K. Rice S. M. (2019). Mental health in elite athletes: increased awareness requires an early intervention framework to respond to athlete needs. Sports Med. Open 5:46. 10.1186/s40798-019-0220-1 31781988 PMC6883009

[B76] Raglin J. S. (2001). Psychological factors in sport performance: the mental health model revisited. Sports Med. 31, 875–890. 10.2165/00007256-200131120-00004 11665914

[B77] Reardon C. L. Hainline B. Miller Aron C. Baron D. Baum A. L. Bindra A.. (2019). Mental health in elite athletes: International Olympic Committee consensus statement. Br. J. Sports Med. 53, 667–699. 10.1136/bjsports-2019-100715 31097450

[B78] Reupert A. (2017). A socio-ecological framework for mental health and well-being. Adv. Ment. Health 15, 105–107. 10.1080/18387357.2017.134290227558710

[B79] Rice S. Walton C. C. Gwyther K. Purcell R. (2021). “Mental health” in Stress, Well-Being and Performance in Sport, eds D. Fletcher and R. Arnold (London: Routledge), 167–187.

[B80] Rice S. M. Gwyther K. Santesteban-Echarri O. Baron D. Gorczynski P. Gouttebarge V.. (2019). Determinants of anxiety in elite athletes: a systematic review and meta-analysis. Br. J. Sports Med. 53, 722–730. 10.1136/bjsports-2019-100620 31097452 PMC6579501

[B81] Rice S. M. Purcell R. De Silva S. Mawren D. McGorry P. D. Parker A. G. (2016). The mental health of elite athletes: a narrative systematic review. Sports Med. 46, 1333–1353. 10.1007/s40279-016-0492-2 26896951 PMC4996886

[B82] Ryan R. M. Deci E. L. (2000). Self-determination theory and the facilitation of intrinsic motivation, social development, and well-being. Am. Psychol. 55, 68–78. 10.1037/0003-066X.55.1.68 11392867

[B83] Ryan R. M. Deci E. L. (2001). On happiness and human potentials: a review of research on hedonic and eudaimonic well-being. Annu. Rev. Psychol. 52, 141–166. 10.1146/annurev.psych.52.1.141 PMID: 11148302 11148302

[B84] Ryff C. D. (2014). Psychological well-being revisited: advances in the science and practice of eudaimonia. Psychother. Psychosom. 83, 10–28. 10.1159/000353263 24281296 PMC4241300

[B85] Sarkar M. Fletcher D. Brown D. J. (2015). What doesn't kill me…: adversity-related experiences are vital in the development of superior Olympic performance. J. Sci. Med. Sport. 18, 475–479. 10.1016/j.jsams.2014.06.010 25035123

[B86] Schinke R. J. Stambulova N. B. Si G. Moore Z. (2017). International society of sport psychology position stand: athletes' mental health, performance, and development. Int. J. Sport Exerc. Psychol. 16, 622–639. 10.1080/1612197X.2017.1295557

[B87] Schinnar A. P. Rothbard A. B. Kanter R. Jung Y. S. (1990). An empirical literature review of definitions of severe and persistent mental illness. Am. J. Psychiatry 147, 1602–1608. 10.1176/ajp.147.12.1602 2244636

[B88] Seery M. D. Holman E. A. Silver R. C. (2010). Whatever does not kill us: cumulative lifetime adversity, vulnerability, and resilience. J. Pers. Soc. Psychol. 99, 1025–1041. 10.1037/a0021344 20939649

[B89] Seligman M. (2011). Flourish: A Visionary New Understanding of Happiness and Well-Being. New York, NY: Free Press.

[B90] Seligman M. (2018). PERMA and the building blocks of well-being. J. Posit. Psychol. 13, 333–335. 10.1080/17439760.2018.1437466

[B91] Stambulova N. B. Engström C. Franck A. Linner L. Lindahl K. (2015). Searching for an optimal balance: dual career experiences of Swedish adolescent athletes. Psychol. Sport Exerc. 21, 4–14. 10.1016/j.psychsport.2014.08.009

[B92] Stambulova N. B. Ryba T. V. Henriksen K. (2020). Career development and transitions of athletes: the International Society of Sport Psychology position stand revisited. Int. J. Sport Exerc. Psychol. 10.1080/1612197X.2020.1737836. Available online at: https://www.tandfonline.com/doi/pdf/10.1080/1612197X.2020.1737836?needAccess=true

[B93] Storm L. K. Henriksen K. Stambulova N. Cartigny E. Ryba T. V. De Brandt K.. (2021). Ten essential features of European dual career development environments: a multiple case study. Psychol. Sport. Exerc. 54:101918. 10.1016/j.psychsport.2021.101918

[B94] Ströhle A. (2019). Sports psychiatry: mental health and mental disorders in athletes and exercise treatment of mental disorders. Eur. Arch. Psychiatry Clin. Neurosci. 269, 485–498. 10.1007/s00406-018-0891-5 29564546

[B95] Sweet P. L. Decoteau C. L. (2018). Contesting normal: the DSM-5 and psychiatric subjectivation. Biosocieties 13, 103–122. 10.1057/s41292-017-0056-1

[B96] Thiel A. Schubring A. Schneider S. Zipfel S. Mayer J. (2015). Health in elite sports – a “Bio-Psycho-Social” perspective. Dtsch. Z. Sportmed. 66, 241–247. 10.5960/dzsm.2015.194

[B97] Turner M. J. Barker J. B. (2013). Resilience: lessons from the 2012 Olympic Games. Int. J. Mutidiscip. Pers. 14, 622–631. 10.1080/14623943.2013.835724

[B98] Uphill M. Sly D. Swain J. (2016). From mental health to mental wealth in athletes: looking back and moving forward. Front. Psychol. 7:935. 10.3389/fpsyg.2016.00935 27445903 PMC4915134

[B99] Van Slingerland K. J. Durand-Bush N. Rathwell C. (2018). Levels and prevalence of mental health functioning in Canadian university student-athletes. Can. J. High. Educ. 48, 149–168. 10.7202/1057108ar

[B100] Wakefield J. C. (2013). The DSM-5 debate over the bereavement exclusion: psychiatric diagnosis and the future of empirically supported treatment. Clin. Psychol. Rev. 33, 825–845. 10.1016/j.cpr.2013.03.007 23706392

[B101] Wakefield J. C. (2016). Diagnostic issues and controversies in DSM-5: return of the false positives problem. Annu. Rev. Clin. Psychol. 12, 105–132. 10.1146/annurev-clinpsy-032814-112800 26772207

[B102] WHO (2004). Promoting Mental Health: Concepts, Emerging Evidence, Practice. Geneva: WHO. Available online at: https://www.who.int/mental_health/evidence/en/promoting_mhh.pdf (accessed June 14, 2021).

[B103] WHO (2006). Constitution of the World Health Organization e-Basic Documents, 45 Edn. Available online at: https://www.who.int/governance/eb/who_constitution_en.pdf (accessed June 14, 2021).

[B104] WHO (2012). WHOQOL Annotated Bibliography. Geneva: WHO Department of Mental Health. Available online at: https://apps.who.int/iris/bitstream/handle/10665/77932/WHO_HIS_HSI_Rev.2012.03_eng.pdf (accessed June 14, 2021).

[B105] Wiese-Bjornstal D. M. (2010). Psychology and socioculture affect injury risk, response, and recovery in high-intensity athletes: a consensus statement. Scand. J. Med. Sci. Sports. 20, 103–111. 10.1111/j.1600-0838.2010.01195.x 20840568

[B106] Wittchen H.-U. Knappe S. Schumann G. (2014). The psychological perspective on mental health and mental disorder research: introcuction to the ROAMER work package 5 consensus document. Int. J. Methods Psychiatr. Res. 23, 15–27. 10.1002/mpr.14008 24375533 PMC6878569

[B107] Wylleman P. (2019). “A developmental and holistic perspective on transitioning out of elite sport,” in APA Handbook of Sport and Exercise Psychology, Vol. 1, Sport Psychology, eds M. H. Anshel, T. A. Petrie, and J. A. Steinfeldt (Washington, DC: American Psychological Association), 201–216.

[B108] Wylleman P. Rosier N. (2016). “Holistic perspective on the development of elite athletes,” in Sport and Exercise Psychology Research: From Theory to Practice, eds M. Raab, P. Wylleman, R. Seiler, A.-M. Elbe, and A. Hatzigeorgiadis (London: Elsevier Academic Press), 269–288. 10.1016/B978-0-12-803634-1.00013-3

